# Zooplankton biodiversity monitoring in polluted freshwater ecosystems: A technical review

**DOI:** 10.1016/j.ese.2019.100008

**Published:** 2019-12-25

**Authors:** Wei Xiong, Xuena Huang, Yiyong Chen, Ruiying Fu, Xun Du, Xingyu Chen, Aibin Zhan

**Affiliations:** a Research Center for Eco-Environmental Sciences, Chinese Academy of Sciences, 18 Shuangqing Road, Haidian District, Beijing, 100085, China; b College of Resources, Environment and Tourism, Capital Normal University, 105 West Third Ring Road, Haidian District, Beijing, 100048, China; c University of Chinese Academy of Sciences, Chinese Academy of Sciences, 19A Yuquan Road, Shijingshan District, Beijing, 100049, China

**Keywords:** Biodiversity, Micro-eukaryotes, Metabarcoding, Freshwater ecosystem, Water pollution

## Abstract

Freshwater ecosystems harbor a vast diversity of micro-eukaryotes (rotifers, crustaceans and protists), and such diverse taxonomic groups play important roles in ecosystem functioning and services. Unfortunately, freshwater ecosystems and biodiversity therein are threatened by many environmental stressors, particularly those derived from intensive human activities such as chemical pollution. In the past several decades, significant efforts have been devoted to halting biodiversity loss to recover services and functioning of freshwater ecosystems. Biodiversity monitoring is the first and a crucial step towards diagnosing pollution impacts on ecosystems and making conservation plans. Yet, bio-monitoring of ubiquitous micro-eukaryotes is extremely challenging, owing to many technical issues associated with micro-zooplankton such as microscopic size, fuzzy morphological features, and extremely high biodiversity. Here, we review current methods used for monitoring zooplankton biodiversity to advance management of impaired freshwater ecosystems. We discuss the development of traditional morphology-based identification methods such as scanning electron microscope (SEM) and ZOOSCAN and FlowCAM automatic systems, and DNA-based strategies such as metabarcoding and real-time quantitative PCR. In addition, we summarize advantages and disadvantages of these methods when applied for monitoring impacted ecosystems, and we propose practical DNA-based monitoring workflows for studying biological consequences of environmental pollution in freshwater ecosystems. Finally, we propose possible solutions for existing technical issues to improve accuracy and efficiency of DNA-based biodiversity monitoring.

## Highlights

• Freshwater ecosystems and associated biodiversity have been highly degraded.
• Biodiversity monitoring is crucial for diagnosing degradation degrees.
• Here we review available methods for monitoring zooplankton biodiversity.
• We propose possible solutions for existing technical issues.

## 1 Introduction

### 1.1 Biodiversity loss in freshwater ecosystems

Since the first proposition of the term ‘biodiversity’ in the United Nations’ Conference on Environment and Development in 1992, the importance of biodiversity has been well recognized and conservation of biodiversity has drawn substantial attention [1]. Among various types of ecosystems, freshwater ecosystems provide unique habitats, supporting a high level of biodiversity. Freshwater ecosystems occupy only approximately 0.8% of the Earth’s surface but support almost 6% of all known species [2]. For example, more than 10,000 fish species live in freshwater ecosystems, accounting for 40% of the known global fish species [3]. In addition, freshwater ecosystems provide irreplaceable goods and services for human beings, such as drinking and irrigation water, food, creation, and regulation of micro-climate [4,5].

However, many factors, particularly those derived from anthropogenic activities such as water pollution and invasive species, have largely degraded freshwater ecosystems over the past several decades [2,6]. Freshwater ecosystems such as rivers and inland lakes are among the most threatened ecosystems on the Earth [4,7]. As a result, biodiversity loss in freshwater ecosystems is much faster than that in terrestrial counterparts [8]. Even worse, biodiversity loss in threaten freshwater ecosystems have not slowed down in recent years [9], despite the fact that great effort has been placed on maintaining or recovering biodiversity in freshwater ecosystems. These efforts have been largely unsuccessful due to frequent disturbance derived from increasing anthropogenic activities and knowledge gaps on biodiversity in freshwater ecosystems [10].

Indeed, biodiversity loss in freshwater ecosystem is likely much more severe than we have realized, as biological response to disturbance in freshwater ecosystems is not completely known, especially on the widespread but hidden microscopic taxa such as zooplankton [3]. A large body of scientific literature has illustrated the biodiversity loss of macro-eukaryotes under human activity disturbance, such as fishes, amphibians, mollusks and crustaceans [[11], [12], [13], [14]]. Nevertheless, studies on biodiversity loss dynamics of micro-eukaryotes are rare [15]. In terms of monitoring and conservation priority, better-known macro-eukaryotes have drawn more attention than micro-eukaryotes such as microscopic zooplankton [16]. Indeed, the highly neglected microscopic zooplankton play vital ecological roles in aquatic food-webs, such as linking phytoplankton and bacteria to high trophic levels such as fish [17]. The protection and recovery of overlooked microscopic zooplankton biodiversity largely determine the conservation of biodiversity at high trophic levels, as well as the integration and functioning of freshwater ecosystems.

### 1.2 Potential indicative roles of zooplankton in freshwater ecosystems

Zooplankton include diverse taxa such as protists, rotifers, copepods and cladocerans, many of which are microscopic [18]. Multiple studies have made a consistent and crucial realization that zooplankton taxa are rapid responders to many environmental stressors, such as hydrological changes, climate changes and anthropogenic activity-induced water pollution [19,20]. Specifically, previous laboratory or field studies have indicated that zooplankton communities were significantly impacted by excessive loading of nutrients [15,19,21], and also negatively affected by microplastics [22], pesticides [23], and pharmaceuticals and personal care products (PPCPs) [24]. As such, researchers have identified their usefulness as ecological indicators to water pollution. For instances, rotifers are used to diagnose ecological impacts of freshwater toxicants, such as endocrine disruptors, bioconcentration of lead, and nanoparticles toxicity [25]. Yang et al. [26] indicated that zooplankton communities could be used to predict ecological thresholds of ammonia nitrogen. Payne et al. [27] listed and recommended seven key reasons for the use of protists as good bio-indicators in aquatic ecosystems. Azevêdo et al. [28] showed that zooplankton communities played a complementary role to macroinvertebrates in indicating variation of the trophic status of waters. Thus, bio-monitoring zooplankton communities has become a widely accepted and irreplaceable aspect in ecological conservation and management of aquatic ecosystems.

### 1.3 Technical difficulties for biodiversity monitoring of zooplankton

Despite rapid development of novel technologies in the past several decades, most traditional morphology-based methods have been used to investigate interactions between biodiversity of zooplankton and environmental pollution in freshwater ecosystems [21,29]. In recent years, several novel strategies, such as metabarcoding (see section 3.1 in this article), represent novel and robust approaches to profile microscopic organisms more efficiently and with finer resolution [30]. Studies have facilitated the use of metabarcoding for assessing diversity of many zooplankton communities [[31], [32], [33], [34]], such as protists [20] and copepods [35]. Metabarcoding-based methods have generated detailed information about biodiversity of zooplankton, providing important support to biodiversity conservation, such as the development of DNA-based ecological status assessment under the European Water Framework Directive [36], and advancement of routine freshwater bio-monitoring by using molecular methods [37].

Yet, there remain many methodological difficulties for biodiversity monitoring of zooplankton in both morphology-based and DNA-based strategies in polluted freshwater ecosystems, and many taxonomic groups of zooplankton are largely undescribed or unknown [2]. The primary issue for morphology-based biodiversity monitoring is resolution and efficiency. When gathering information on zooplankton composition and abundance in a traditional way, it relies heavily on taxonomists who identify specimens under a binocular microscope. The process is time- and cost-consuming and requires high expertise. Moreover, both the number of samples that can be analyzed and the availability of competent taxonomists are limited. Meanwhile, relatively low resolution imposes limitations on species identification, particularly at larval/juvenile stages and for cryptic species or species complex. In addition, morphology-based methods are challenged by rare species detection [31], which is of great significance for biological conservation. Rare species, which are often either endangered species or species under severe environmental stresses in polluted ecosystems, should be protected or analyzed in priority.

Such difficulties in accurate morphological identification of species not only impede biodiversity monitoring in a traditional way, but also hinder biodiversity investigation by using DNA-based approaches [31,38]. Despite that DNA-based methods obviously advance the efficiency and resolution, particularly for studies aiming at large geographical scales, the precondition for DNA-based methods relies on available reference databases that must be constructed based on morphological identification [39]. In addition, there are challenges in quantification of taxon abundance in using DNA-based methods such as metabarcoding, as metabarcoding analysis has not shown good agreement between specie abundance data and sequence abundance (e.g., Ref. [40]).

### 1.4 Our aims

To advance management of impaired freshwater ecosystems, this review aims to provide an overview of available methods for monitoring biodiversity of eukaryotic zooplankton, including both morphology-based and DNA-based approaches. In addition, we discuss technical difficulties, as well as causes and consequences of these difficulties in biodiversity monitoring. Finally, we propose a practical monitoring workflow to study biological consequences of environmental pollution in aquatic ecosystems as take-home information.

## 2 Morphology-based methods for zooplankton biodiversity monitoring

### 2.1 Traditional morphological methods

**Fig. 1:**
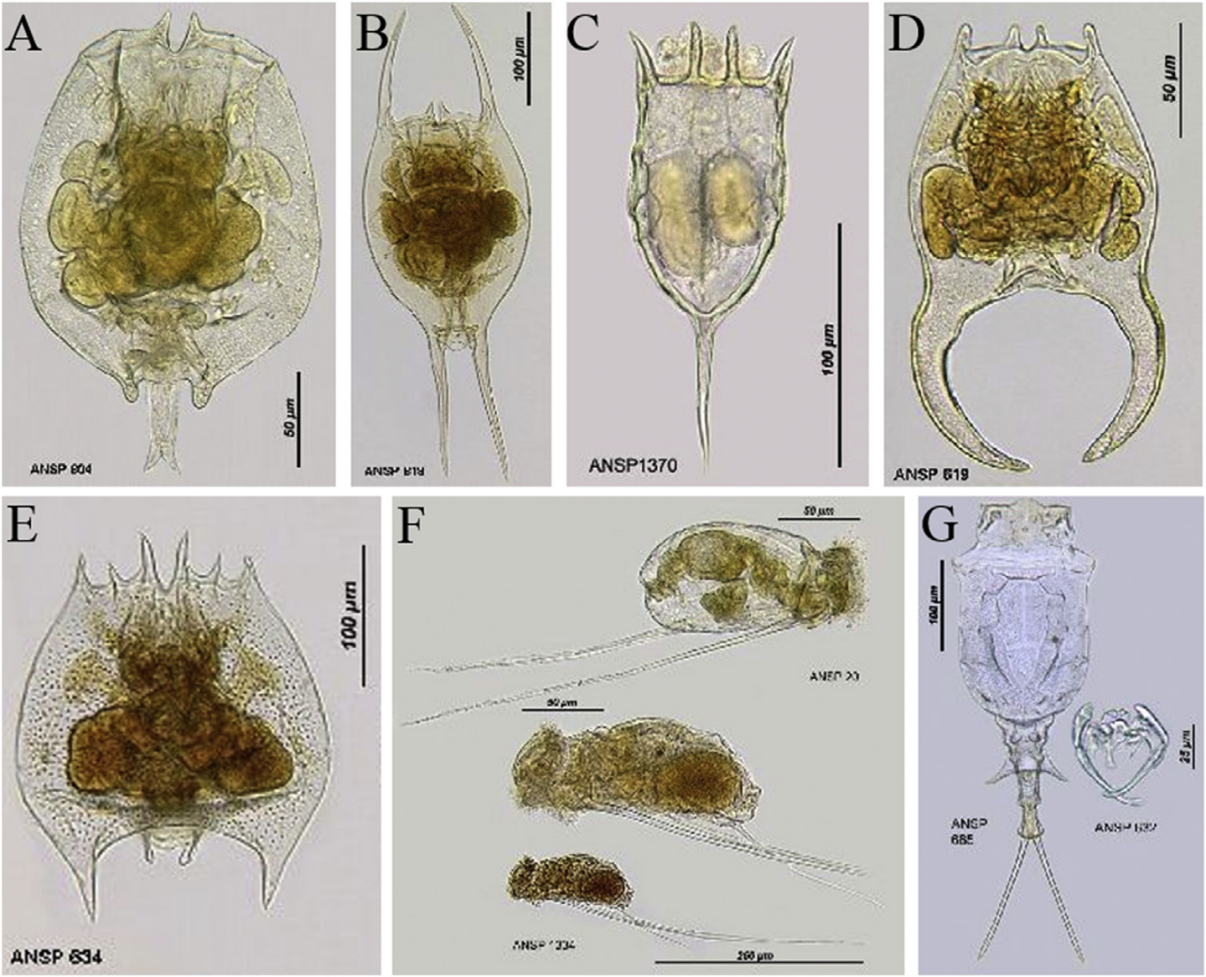
Photos of zooplankton observed by traditional morphological methods referred from Rotifer World Catalog [46]. A: *Brachionus angularis* Gosse, 1851; B: Brachionus diversicornis (Daday, 1883); C: *Keratella cochlearis* (Gosse, 1851); D: *Brachionus forficula* Wierzejaki, 1891; E: *Brachionus quadridentatus* Hermann, 1783; F: *Keratella cochlearis* (Gosse, 1851); G: *Trichotria tetractis* (Ehrenberg, 1830).

The acquisition of biodiversity data has historically been based on morphological characterization of species. Researchers use many tools such as nets, pumps or water bottles to collect specimens and gatherer formation of composition and abundance of species, and then collected specimens are subjected for identification by taxonomists under a microscope [[41], [42], [43]]. This traditional technique is considered to be useful for the identification and enumeration of microplankton (Fig. 1), and thus providing invaluable information on species identification [41,42]. For instance, Humes [44] have reported approximate 11500 morphological species of copepods. Kreutz & Foissner [45] edited a book of protozoological monographs, recording 670 species of protists, micro-metazoans and bacteria in ponds of Simmelried in Germany.

**Fig. 2:**
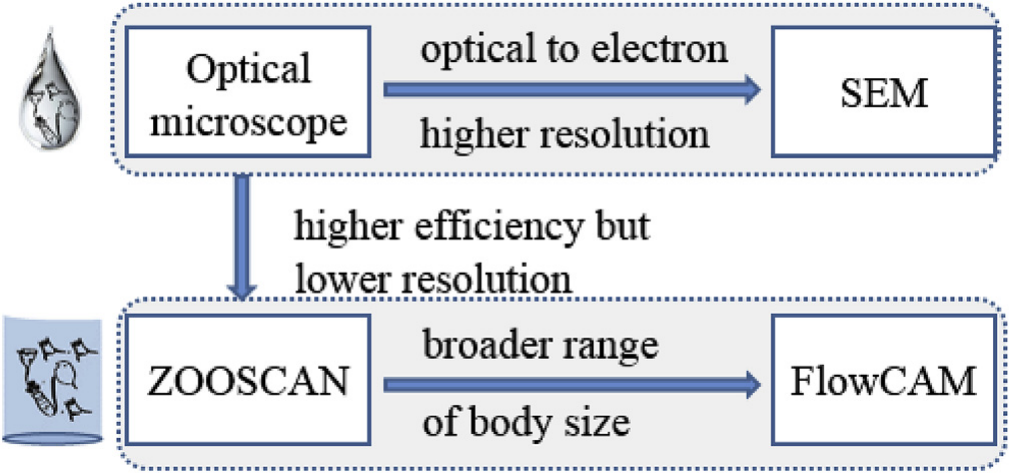
Morphology-based methods for zooplankton biodiversity monitoring.

With the advent of morphological identification by electron microscopes, the scanning electron microscope (SEM) has become a powerful analytical tool (Fig. 2). The use of SEM significantly increases the resolution in specimen identification and helps detect taxonomy keys. For instance, by using SEM, several rotifer species with differentiated trophi were identified in an apparently morphological uniform genus [47]. Papa et al. [48] updated taxonomic status of crustacean zooplankton in a lake in Philippines using a combination of light and scanning electron microscopes. Hines [49] studied the biogeography of freshwater ciliates (protozoa) in Florida, USA with a high resolution of species identification by SEM. Thus, SEM has become an important complementary tool for morphology-based monitoring of zooplankton, particularly on microscopic taxa with debates.

### 2.2 New morphological identification methods

It is obvious that both traditional microscopes and SEMs can detect biological features in detail but fail to analyze a large number of species rapidly. With the advent of digital imaging and scanning technologies, several automatic or semiautomatic instruments have been developed to rapidly analyze zooplankton samples. The two representative ones are ZOOSCAN system [41] and Flow Ctometer And Microscope (FlowCAM) [42]. ZOOSCAN takes digital images of zooplankton samples and analyze images based on available databases in a fast and (semi-)automatic way. By integrating ZooProcess and Plankton Identifier software, ZOOSCAN system can acquire and classify digital zooplankton images from collected zooplankton communities by comparing scanned images of each organism with a reference library. Meanwhile, the biomass and accurate body size of each species can be estimated by ZOOSCAN system for a quantitative study of zooplankton samples. However, ZOOSCAN is suitable for organisms with body size ranging from 200 ​μm to several centimeters, which excludes most taxa of rotifera and protozoa zooplankton in freshwater ecosystems. Currently, ZOOSCAN systems are popularly applied in marine ecosystems to rapidly estimate biomass and size distribution of mesozooplankton for biodiversity monitoring [50].

FlowCAM, which is developed based on the fluid imaging technology, is an automated imaging flow cytometer. FlowCAM uses laser detection to capture digital images of particles/organisms in a fluid. Image analysis of collected digitized images enable accurate estimation of the abundance and size of organisms and automatic classification of organisms [51]. Depending on the setup of the instrument, FlowCAM can detect organisms with body size from 3 to 3000 ​μm, including almost all species in zooplankton communities in freshwater ecosystems, especially in polluted ones dominated by smaller-sized species [15]. Though FlowCAM was created for analyzing phytoplankton [52,53], more studies have used advanced FlowCAM to quantitatively analyze zooplankton [[54], [55], [56]]. Ide et al. [57] carried out an adequate evaluation of zooplankton and phytoplankton in feeding of two omnivory copepod species using FlowCAM. Stanislawczyk [58] demonstrated the capability of FlowCAM to distinguish between closely related zooplankton taxa and detect rare species such as exotic species at early developmental stages. Wang et al. [59] used FlowCAM to quantitatively monitor zooplankton grazers in low abundance in algal cultures for early warning. In addition, Wong et al. [60] optimized FlowCAM to overcome difficulties in low abundance samples. Altogether, the ability to automatically detect and count zooplankton in mixed-species samples in a rapid way is an obvious advantage in monitoring biodiversity in polluted freshwater ecosystems. In addition, FlowCAM can detect low abundance species, such as threatened or newly introduced nonnative invasive species, for conservation management and risk assessment.

### 2.3 Technical challenges

Traditional freshwater biodiversity monitoring is often based on direct observation methods, which are usually done by using an instrument to directly (e.g., microscope or SEM) or indirectly (e.g., ZOOSCAN and FlowCAM) observe morphological features of zooplankton organisms. There is no doubt that such methods cannot be totally replaced by new strategies such as genetic methods, thus we should know technical issues that have not been solved to possibly avoid errors.

By using SEM, the accuracy of traditional morphological approaches can be highly improved [61]. Obviously, the detailed features, particularly new taxonomic keys, should be deeply investigated and validated by competent taxonomists. Such detailed investigations can subside debates on taxonomy but are unlikely appropriate for large-scale surveys for ecological studies, such as causes and consequences of environmental pollution in freshwater ecosystems. ZOOSCAN and FlowCAM highly increase the efficiency of sample processing by automating and digitalizing samples. However, both ZOOSCAN and FlowCAM compromise resolution of species identification [41,42]. Images captured by ZOOSCAN or FlowCAM are usually identified to high taxonomic levels such as genus or above [42,62]. In addition, ZOOSCAN has sound performance for species with body size ranging from 200 ​μm to several centimeters. While in polluted freshwater, zooplankton communities are commonly dominated by small organisms such as rotifers and ciliates [15,63]. Meanwhile, libraries for taxonomy classification of digital images were constituted from zooplankton species in oceans, thus great effort is needed to create reference libraries derived from freshwater ecosystems. FlowCAM is more compatible to monitoring zooplankton in polluted freshwater ecosystems, but it is also limited by the same problems as ZOOSCAN (i.e., reference libraries). In addition, automatic classification of samples is error-prone, as all types of objects, including artifacts, are encountered in collected zooplankton samples [42,51,64]. As a result, validation or data quality control must be done to avoid errors generated from automatic processes.

## 3 DNA-based methods for zooplankton biodiversity monitoring

### 3.1 Biodiversity monitoring based on (meta)barcoding

To overcome the limitation of conventional morphology-based methods, improved methods are urgently required for more rapid, sensitive and efficient detection of zooplankton species in freshwater ecosystems. With the development of molecular biology and sequencing technologies, DNA-based approaches provide opportunities for species identification and biodiversity survey in complex communities [33,65,66]. Taxa can be distinguished by a specific short DNA sequence derived from several evolutionarily conserved genes in genomes or organelles (i.e., DNA barcode) [67]. Thus, DNA barcoding-based method is particularly powerful for identifying cryptic species by PCR amplification and sequencing of the selected DNA barcodes, which can potentially avoid artificial errors by traditional methods. Recently, technical advances of high-throughput sequencing (HTS) have largely revolutionized DNA barcoding-based methods, allowing rapid species identification of individuals across diverse ranges of taxa in environmental samples simultaneously in a single effort (i.e., DNA metabarcoding) [68].

When compared to traditional methods, DNA metabarcoding-based methods are minimally invasive, faster, comprehensive, and time/cost-effective [38,[69], [70], [71]]. As a result, DNA metabarcoding-based methods have been popularly applied in the detection and bio-monitoring of biodiversity in a wide range of taxonomic groups, such as sharks, fishes, amphibians, and insects in aquatic ecosystems [70,[72], [73], [74]]. Shaw et al. [75] compared fish communities detected by DNA metabarcoding with that by conventional fyke netting in the North Para River (NPR) in South Australia, and their results showed that DNA metabarcoding approach could detect 100% of the net-caught fish species under the premise that the sampling strategies were appropriate. The number of detected fishes *via* DNA metabarcoding were higher than that by traditional methods clearly indicating that DNA metabarcoding has the potential to become an important approach in biodiversity monitoring in freshwater ecosystem. In addition, Yang et al. [76] compared the detection capacity of DNA metabarcoding with traditional morphology-based methods in studying the zooplankton community structure in eutrophic Lake Tai Basin of China, and their results showed that the species composition and biomass represented by sequence read counts obtained by DNA metabarcoding were consistent with morphological data. Indeed, DNA metabarcoding-based methods largely facilitated our understanding of biological consequences of environmental pollutions in freshwater ecosystems. Li et al. [77] detected multiple taxa in protist and metazoan communities in the Yangtze River Delta using DNA metabarcoding, and they revealed that the community structure and biodiversity changes were mainly driven by nutrient levels. Xiong et al. [15,63] illustrated that large-sized arthropods in slightly polluted river segments were shifted to small-sized species such as rotifers and ciliates in highly disturbed areas, clearly putting forward an important ecological mechanism that the role of species sorting can override dispersal as the dominant driver in determining community structure (i.e., fine-scale species sorting hypothesis). Therefore, DNA metabarcoding-based methods offer a sensitive, fast and powerful strategy to investigate biodiversity fluctuation dynamics and further demonstrate important ecological problems caused by water pollution derived from human activities [15,78].

**Fig. 3:**
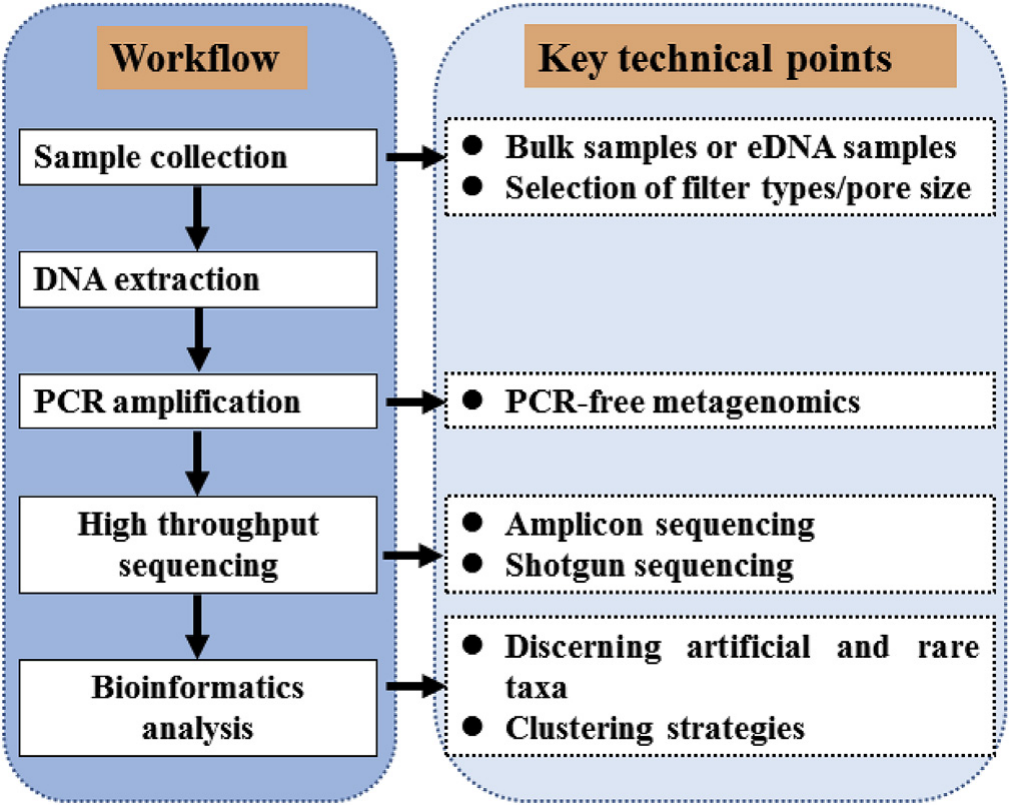
Workflow and key technical points of metabarcoding in biodiversity monitoring of zooplankton.

The typical operational procedure of DNA metabarcoding technology for freshwater biodiversity monitoring consists of the following five steps: sample collection, DNA extraction, PCR amplification for selected genetic markers, sequencing and bioinformatics analysis (Fig. 3). Here we briefly discuss the key technical issues that require attention to avoid or decrease errors/bias during biodiversity assessment. It should be noted that despite DNA metabarcoding method has been widely used in biodiversity research and monitoring [68,79,80], many technical issues have not solved or well solved [29, 38]. Thus, perfect and standardized protocols are still largely needed [38,81].

#### 3.1.1 Sample collection and DNA isolation/capture

There are two strategies of sampling zooplankton in biodiversity monitoring. One is sampling bulk specimen samples (community DNA metabarcoding), by which zooplankton can be quantitatively collected and enriched by filtering the same volume of water at each sampling site using plankton nets [[15], [26], [63]]. The collected samples can be directly preserved in anhydrous alcohol, or further filtered through 5-μm microporous filter membranes, then stored at −20 ​°C for subsequent DNA extraction. The other strategy is sampling environmental DNA samples (eDNA metabarcoding) [72,82]. Environmental DNA (eDNA) is the suspended DNA traces from skin cells, organelles, gametes or even extracellular DNA of all organisms living in aquatic ecosystems [68]. Environmental DNA can be captured onto filter membranes by filtering 1-2 ​L of water in field or laboratory and preserved in anhydrous alcohol. To date, there is still a debate on the selection of filers and associated parameters including type, pore size, pre-filtration and filter preservation strategy during the eDNA capture process. The most commonly used filter for eDNA capture is 0.45-μm cellulose nitrate membrane, followed by 0.75/1.2-μm glass microfiber filers or polyethersulfone [72,81]. Besides, in order to avoid false negatives caused by random sampling of rare taxa, it is important to conduct repeated sampling for at least three times at each sampling site [83].

It is important to keep in mind when we collect eDNA samples for biodiversity monitoring that 1) the stability, dispersal and degradation rate of eDNA are substantially influenced by various environmental factors such as temperature, pH, light intensity and water chemistry [79,84]; 2) the temporal and spatial distribution of eDNA highly vary in different ecosystems such as rivers and lakes, largely owing to the transport of eDNA over large geographical scales in rivers and long retention time in lakes [[85], [86], [87]]; 3) eDNA from different vertical depth of river and lakes has different biological significance, with that of surface water indicating recent site biodiversity while sediments representing historical biodiversity [68]. Thus, steps of eDNA capture need to be optimized in each study, especially for highly polluted water with special hydrochemical features.

Currently, most studies relating to zooplankton biodiversity analysis used bulk specimen samples [15,63,78]. However, when using eDNA to perform biodiversity monitoring, sampling will be easier and less invasive [88]. assessed the potential of eDNA metabarcoding to monitoring zooplankton biodiversity, by comparing method of eDNA metabarcoding to bulk sample metabarcoding and morphological methods, and they suggested that eDNA metabarcoding should be able to provide complementary insights in biodiversity monitoring of zooplankton.

#### 3.1.2 Selection of robust genetic markers and universal primers

“DNA metabarcode” is a fragment of DNA sequence being used for taxonomic identification of multiple species existing in a mixed sample collected from communities or environments [68]. The selection of DNA metabarcode will greatly influence the detected species number and composition of taxonomic groups, as well as the accuracy of species identification [89]. Thus, the choice of appropriate genetic makers is of vital importance for DNA metabarcoding analysis [90]. A desired genetic marker for PCR primer design is expected to simultaneously possess both evolutionarily conserved and hypervariable regions, of which conserved regions were used to design universal primers for amplifying a wide range of species or taxonomic groups (versatility), and hypervariable regions in the amplicons were used to accurately distinguish the closely related species (high resolution) [90].

Owing to the relatively high evolutionary rate and high taxonomic resolution capability, the mitochondrial cytochrome *c* oxidase subunit I gene (COI) is considered as a competent candidate genetic marker for metabarcoding survey for metazoa [43,90,91]. However, the amplification of COI in some taxonomic groups such as crustaceans is still a difficult task, particularly in bulk samples (see the review by Ref. [90] and references therein). To find a desired genetic marker to metabarcode zooplankton biodiversity, Zhan et al. [[31], [92]] designed primer pairs based on 18S and compared three commonly used genetic marker, including 18S, COI and 16S by testing performance of different primer pairs, and they proposed that 18S was the most efficient and powerful genetic marker for zooplankton biodiversity monitoring. Clarke et al. [93] claimed that COI [94] and 18S [31] recovered similar taxonomic coverage and beta-diversity, but different biomass and sequence reads relationship and resolution, by comparing performance of these two best-known primer sets in biodiversity study of zooplankton. Yet, Andújar et al. [43] argued that COI still should be the most appropriate DNA metabarcode for bulk community analysis based on the following reasons: 1) high resolution for taxonomic identification; 2) extensive coverage of COI in reference sequence databases; 3) advantage of a protein-coding gene to identify spurious sequences.

For eDNA metabarcoding, there are more challenges for selecting genetic markers and primers, mainly due to low quality and quantity of eDNA and high proportion of co-amplification of microbial DNA [95]. Here, we urgently suggest that genetic markers and associated universal primers should be well tested before application, particularly on several parameters such as coverage of taxonomic groups and amplification efficiency and bias. It is true that there are no perfect genetic markers and associated universal primers for all taxonomic groups, but careful consideration of all influential parameters can largely improve the power of DNA metabarcoding-based biodiversity survey.

While researchers struggle to select efficient genetic marker and primer sets in metabarcoding, PCR-free methods are emerging recently, such as shotgun sequencing after mitochondrial enrichment [96]. This method is independent on PCR, as mitochondrial DNA is enriched during metaDNA extraction and sequenced by ultra-deep high-throughput sequencing (HTS), and then COI sequences are retrieved by bioinformatic analyses [96]. To avoid high cost of ultra-deep HTS and advance PCR-free methods in routine environmental biomonitoring, a gene-enrichment technique was recommended by Dowle et al. [97]. This method is derived from probe enrichment techniques used in human genomic research and uses gene capture probes to enrich target genes, followed by HTS to monitor biodiversity of aquatic communities [97]. These two methods overcome PCR-derived biases to provide better estimate of species abundance or biomass of target organisms, enhancing both the DNA sequence information per taxon and the number of taxa.

#### 3.1.3 Bioinformatics analysis

Enormous short sequence reads are generated by high throughput sequencing (HTS) in DNA metabarcoding analysis, particularly after processing a large number of samples collected from large geographical scales. Such big data sets pose vast challenges to subsequent data processing procedures. A general data analysis process includes raw data filtering, clustering of operational taxonomic units (OTUs), and taxonomy assignment.

Available evidence shows that sequencing errors greatly influence the biodiversity estimates [38,98]. Thus, the removal of sequencing errors is the first and one of the most important steps, which is challenged by discerning real rare taxa from sequencing errors. Both rare species and artificial reads are usually recovered by low number of sequences (e.g., low abundance OTUs), and the process of removal artificial reads is prone to filter out a large proportion of rare species in samples [99]. Depending on research aims, the clustering strategy for OTUs is another essential step for assessing community biodiversity using DNA metabarcoding. These clustered molecular OTUs were assigned to traditional taxonomic species in biodiversity monitoring, while predefined similarity threshold (such as 97%) used in the clustering step may split one traditional species into two or more OTUs or sequences for different taxa were clustered into the same OTUs. To resolve this issue, newly developed methods, such as DADA2 [100] and UNOISE3 [101] were advanced algorithms of discerning and removing sequencing errors and picked OTUs at similarity threshold of 100%, and LULU was created for removing erroneous OTUs [102]. Xiong & Zhan [34] tested different clustering strategies in studying zooplankton diversity, including newly developed non-clustering (DADA2 and UNOISE 3) and clustering with different thresholds. Their results showed that largely varied alpha biodiversity estimates were obtained by different clustering strategies, but the ecological conclusions were consistently drawn by non-clustering and clustering thresholds of 97–99%.

### 3.2 Indicator species detection based on metabarcoding

Owing to high sensitivity to environmental stressors, biological indicator species play crucial roles in assessing pollution effects in aquatic ecosystems and provide early warning of environmental changes [15,63,103]. Identifying indicator species, rather than traditional qualitative analysis of all species in biological communities, provides a convenient and appealing approach in environmental monitoring, conservation and management [104]. DNA barcoding has enhanced the sensitivity and efficiency of species-level identification and highlights its wide use in indicator species detection in biodiversity monitoring efforts [67,105]. A number of studies have documented the utility of DNA barcoding for species identification, such as fish [106], copepods [107], crustaceans [108], and molluscs [109]. Recently, the application of DNA barcoding method has been extended dramatically owing to the high-throughput sequencing (HTS), enabling the identification of indicator species from mixed species samples or communities [[110], [111]]. Using HTS technologies to study the relationship between geographical distributions of bacterial communities and water pollution, Yang et al. [112] revealed several effective bacteria as bio-indicators to assess river pollution in Songhua River, suggesting the potential of micro-organisms as useful biological indicators. Metabarcoding-based methods have been used to identify a variety of aquatic indicator species for water-quality assessment and biodiversity monitoring, such as arthropods [[63], [83], [104]], gastropods [113], amphibians [83,114], reptiles [115], fishes [116,117], and mammals [104,118]. Thus, metabarcoding-based methods are robust tools to identify indicative species from microscopic zooplankton.

### 3.3 Rare species detection based on metabarcoding and qPCR

Typically, aquatic communities are composed of a few dominant species and a high diversity of low abundance species (i.e., ‘‘rare biosphere’’, [119,120]). The rare biosphere taxa may switch to new abundant members of a community in response to environmental perturbation or change, and thus could provide a potential resource of genetic and functional diversity to maintain ecosystem functioning. The rare biosphere in running water ecosystems mainly include two types of species: native rare species and newly introduced non-indigenous species (NIS). Owing to environmental pollution or demographic stochasticity, native rare species may be at risk of extinction and become endangered [121]. In addition, newly introduced NIS generally maintain low population density in communities for a long period of time, even in cases when they eventually become dominant to cause large-scale negative effects [31]. Therefore, novel methods and effective measures should be taken to explore and monitor the rare biosphere, in particular to identify native threatened rare species for conservation and recently introduced NIS for biosecurity. However, there remain major technical challenges to detect rare species, especially for microscopic organisms in freshwater ecosystems, owing to their tiny body size, diverse species, complex community structure, and ambiguous morphological traits [90,122].

Metabarcoding has provided effective access to investigating rare species in complex aquatic communities [38,123]. Zhan et al. [31] quantitatively tested the sensitivity of metabarcoding and claimed that metabarcoding enabled the detection of rare species in complex zooplankton communities (biomass as low as 2.3 ​× ​10^−5^%). Mounting studies highlight the crucial role of metabarcoding in monitoring endangered species for conservation (see the review by Ref. [124] and references therein). Meanwhile, metabarcoding helps early detection of NIS in aquatic ecosystems. For instance, Brown et al. [125] detected 24 non-indigenous species in Canadian ports, 11 of which were firstly reported in the locations. Westfall et al. [126] proposed a metabarcoding-based new approach to molecular biosurveillance of invasive zooplankton species. Besides, for accurately identifying protected species, DNA metabarcoding-based methods can provide useful information about speciation time and phylogenetic diversity to perform quantitative comparison rather than simply depending on the number of species within this area [127]. In addition, commonly used genetic markers for HTS, such as COI, mt16S, 18S rDNA, would largely increase the comparability among different species, allowing us to make the quantitative measurement of genetic diversity and evolutionary potential of rare species and to confirm the relationships between endangered species and their surrounding species. Thus, metabarcoding provides important insights in rare species detection, which allows managers to make scientific decisions of species, populations and individuals for protection.

Except screening rare species in mixed complex communities using metabarcoding, species-specific PCR methods, including conventional PCR (cPCR) and real-time quantitative PCR (qPCR), are DNA-based approaches to directly detect targeted endangered or NIS species in low abundance [128]. Conventional PCR has been applied to the detection of a variety of aquatic NIS, including amphibians (*Rana catesbeiana* [129], crustaceans (*Procambarus clarkii* [130], mollusca (*Limnoperna fortune,* [128], and many other taxa. Real-time quantitative PCR (qPCR), which is usually considered to be more sensitive than cPCR, provides a promising molecular tool for detection and quantification of rare species [117,131]. For example, a quantitative real-time PCR (qPCR) amplification of 16s rDNA was performed to quantitatively assess harmful algal bloom (HAB) risks in the Great Lakes [132]. Recently, novel solutions, such as carbon nanotube platforms [133], nucleic acid sequence-based amplification (NASBA [134], and droplet digital PCR (ddPCR) [135], have extended the use of DNA-based methods in monitoring targeted rare species.

### 3.4 Technical difficulties and possible solutions

**Fig. 4:**
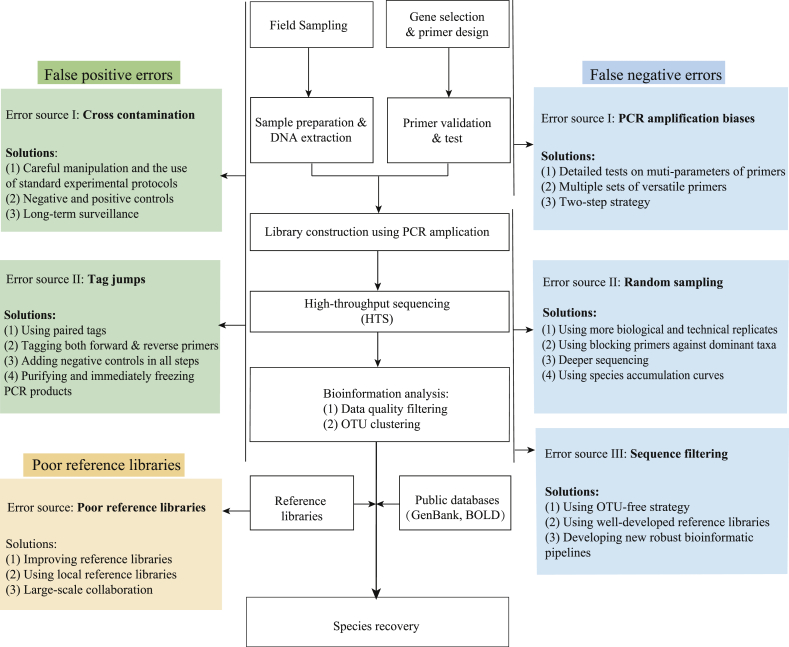
Technical difficulties and possible solutions in flowchart of biodiversity monitoring of zooplankton.

Although DNA-based methods exhibit evident advantages in assessing biodiversity, there remain technical difficulties and challenges that need to be addressed for the implementation of biodiversity monitoring programs. As mentioned above, PCR-free approaches are able to overcome some limitations of PCR-based methods. However, these methods have not been well tested in complex real-community samples [96] and showed low sensitivity to detect rare species [[90], [136]]. In this study, we focus on the commonly used PCR-based methods (i.e., DNA metabarcoding, Fig. 4).

#### 3.4.1 False positives and negatives

The most notable problem confronting the process of DNA metabarcoding analyses is the risk of error sources, including false positives (species detected but absent) and false negatives (species undetected but present). A variety of issues can lead to false positives and negatives [38,90,137].

False positives, which is also known as Type II errors, can result from cross-contamination from multiple sources, such as improper handling of samples, inadequate specificity primers, and errors in data analysis [38,77,124]. As a result of the increasing throughput of sequencing technologies, the pooling of samples may lead to cross-contamination [138]. Careful manipulation, standard experimental protocols and contamination removal pipelines may reduce cross-contamination risks and false positives. Moreover, negative and positive controls should be added in the entire process [[83], [111], [116], [139]]. In addition to cross-contamination, tag jumping among pooled samples is another reason for false positives [[38], [90], [140]]. There are some causes of tag jumping for HTS-based studies, such as remaining unused tagged primers, using only one tagged primer, or sequencing long amplicons [38]. To minimize the probability of false positives caused by tag jumping, paired tags should be added at both sides of amplicons [140]. Increasing the number of biological replicates, adding negative controls and applying strict filtering pipelines would provide effective approaches to reducing the effect of tag jumping [141]. In addition, other approaches that can contribute to avoidance of tag jumping includes carefully purifying PCR products, immediately freezing PCR products, and shortening their storage time [[29], [38], [141]].

The sources of false negatives (Type I errors) are mainly derived from random sampling and biased PCR amplification [[29], [90]]. When investigating community structure with DNA metabarcoding, random sampling processes can result in low reproducibility among replicate samples, especially for rare species, thus leading to false negatives [99,142,143]. Several technical approaches should be performed to decrease false negatives by using more biological and technical replicates, deeper sequencing, primers blocking against dominant taxa and optimized species occupancy models [38,[90], [144]]. Another common and severe problem influencing taxonomic resolution power in DNA metabarcoding studies is PCR amplification biases among species [29,38]. This error source can be introduced by many factors, such as universality of the primers, annealing temperature, the number of replication cycles, and relative abundance of different species in a community [143,145]. PCR amplification bias is strongly influenced by the annealing temperature and the number of replication cycles during library preparation. Low annealing temperature and cycle number can reduce the bias [146]. Additional efforts include optimizing conditions of PCR amplification by increasing template concentrations and adopting wise primers [147,148]. More importantly, the utilization of multiple versatile primers or two-step nested PCR would reduce amplification biases and enhance the opportunity of rare species detection [38,40,82].

#### 3.4.2 Reference database

Retrieving the taxonomic composition from sequences data is one of the primary aims when we monitor biodiversity using DNA-based methods. This step is completely dependent on comparing sequences against a metabarcode reference dataset containing taxonomy information [111]. The high-quality and complete reference databases for taxonomic assignment directly determine the reliability of DNA metabarcoding. Even though a large number of DNA sequences has been submitted to public databases, unbalanced representation of different taxonomic groups in these public sequence databases is a major issue [39]. Available sequences for poorly studied species in public databases remain low when compared to commercially important or better studied species [39]. Thus, broad collaborations and sharing among different research groups who particularly have their own taxa of interest, should be made to develop and improve community-level reference libraries [70]. Additionally, most sequence information was obtained from traditional molecular makers, such as COI and ribosomal RNA genes in these databases, which is not enough to identify complex communities and closely related species. Thus, more sequence information with diverse molecular makers should collect in order to build comprehensive reference libraries.

From a taxonomic point view, reference datasets (Table 1) are poorly connected, though part of them, such as EMBL, GenBank and DDBJ, have been united into the International Nucleotides Sequence Database Collaboration (INSDC) and synchronized daily [111]. Lacking coherence among datasets largely limits meta-analyses on metabarcoding-based studies. Thus, collaborative efforts remain necessary in the future for biodiversity monitoring at the global scale.

**Table 1:** The most recent and important reference databases for zooplankton.

Database name	Organisms covered	Genetic markers	Established year	Website	Reference
EMBL-EBI	Bacteria/Archaea, Eukaryota	16S/23S rRNA 16S/18S/COI	1980	https://www.ebi.ac.uk/ena	--
GenBank	Bacteria/Archaea, Eukaryota	16S/23S rRNA 16S/18S/COI	1982	https://www.ncbi.nlm.nih.gov/genbank/	[149]
DDBJ	Bacteria/Archaea, Eukaryota	16S/23S rRNA 16S/18S/COI	1987	https://www.ddbj.nig.ac.jp/index-e.html	[150]
SILVA	Bacteria/Archaea, Eukaryota	16S/23S 18S/28S	2007	https://www.arb-silva.de/	[151]
BOLD	Animals, Plants, Fungi	COI rbcl, matk ITS	2007	http://www.boldsystems.org/	[152]
PR2	Protists	18S	2012	ssu-rrna.org/pr2	[153]

## 4 Future perspectives

In summary, metabarcoding is revolutionizing the investigation of freshwater biodiversity and presents a powerful tool of detection of “hidden diversity” under water surface [154]. We propose that DNA-based metabarcoding should be embraced by researchers and managers, as its inherent practicable and applicable features can largely solve technical issues in biodiversity assessment in polluted freshwater ecosystems. Indeed, DNA-based metabarcoding has been approved by several international organizations, such as the European Water Framework Directive of European Union. Yet, many technical issues still remain in the application of metabarcoding-based biomonitoring in freshwater ecosystems. We should firstly continue the development of taxonomically comprehensive reference databases based on multiple genetic markers to support taxonomic retrieval of metabarcoding analyses. Additionally, we should screen more variables gene regions, which can ensure correct identification, discrimination and detection of closely related or cryptic species. Alternatively, as HTS becomes more accessible and less expensive, the use of short-gun sequencing strategy to sequence meta-DNA extracted from bulk samples would increase the resolution of taxonomical identification. In addition, shot-gun sequencing is a PCR-free method, which is beneficial to quantification of taxon abundance. Spiking the standard DNA into different samples is a good method to make advance of quantification in metabarcoding analyses. Finally, there is no doubt that traditional morphology-based methods cannot be discarded, although many technical issues still exist among morphology-based methods. Both traditional morphology-based and DNA-based methods must be cross-referenced to ensure accurate and rapid identification of zooplankton species, and to promote the dissection of causes and consequences of biodiversity loss in polluted freshwater ecosystems.

## Author contributions

A.Z., B.S. and W.X. conceived the study. All authors wrote and revised the manuscript.
